# Efficacy and safety of platelet-rich plasma injections for the treatment of osteoarthritis: a systematic review and meta-analysis of randomized controlled trials

**DOI:** 10.3389/fmed.2023.1204144

**Published:** 2023-06-27

**Authors:** Yongqing Xiong, Cheng Gong, Xumiao Peng, Xianlei Liu, Xinda Su, Xi Tao, Ying Li, Youliang Wen, Wei Li

**Affiliations:** Gannan Medical University, Ganzhou, Jiangxi, China

**Keywords:** platelet-rich plasma, osteoarthritis, systematic review, meta-analysis, randomized controlled trials

## Abstract

**Background:**

In recent years, platelet-rich plasma (PRP) injections for osteoarthritis (OA) have been widely promoted in clinical practice, but their effectiveness is controversial. Therefore, we conducted a meta-analysis of relevant randomized controlled trials (RCTs) to determine the efficacy and safety of PRP injections for the treatment of OA.

**Methods:**

We searched databases including Embase, Web of Science, Medline, PubMed, and the Cochrane Library for relevant studies. Two researchers (YQX and CG) performed literature screening, baseline data extraction, literature quality assessment, and heterogeneity analysis of RCTs from the retrieved studies. Based on the magnitude of heterogeneity *I*^2^, random-effects or fixed-effects models were selected for the meta-analysis.

**Results:**

We included 24 RCTs comprising 1344 patients with OA who met the inclusion criteria, with the main types of morbidity being knee osteoarthritis (KOA), hip osteoarthritis (HOA), ankle osteoarthritis (AOA), and temporomandibular joint osteoarthritis (TMJOA). Our results indicate that PRP injections were effective in improving Visual Analog Scale (VAS) pain scores in patients with KOA, HOA, and AOA compared to controls (AOA, MD = −1.15, CI = 95% [−1.74, −0.56], *I*^2^ = 40%, *P* < 0.05; KOA, MD = −1.03, CI = 95% [−1.16, −0.9], *I*^2^ = 87%, *P* < 0.05; TMJOA, MD = −1.35, CI = 95% [−1.74, −0.97], *I*^2^ = 92%, *P* < 0.05) but showed no significant efficacy in patients with HOA (MD = −0.27, CI = 95% [−0.8, 0.26], *I*^2^ = 56%, *P*>0.05). Compared to controls, PRP injections were effective in improving Knee Injury and Osteoarthritis Outcome Score (KOOS), including the patient's pain symptoms, activities of daily living (ADL), and adhesion symptomatology, but not for that of sports function (KOOS-pain, MD = 2.77, CI = 95% [0, 5.53], *I*^2^ = 0%, *P* < 0.05; KOOS-symptoms, MD = 3.73, CI = 95% [0.76, 6.71], *I*^2^ = 0%, *P* < 0.05; KOOS-ADL, MD = 3.61, CI = 95% [0.79, 6.43], *I*^2^ = 0%, *P* < 0.05; KOOS-QOL, MD = 4.66, CI = 95% [0.98, 8.35], *I*^2^ = 29%, *P* < 0.05, KOOS-sport, MD = 0.48, CI = 95% [−3.02, 3.98], *I*^2^ = 0%, *P* > 0.05). PRP injections were effective in improving Western Ontario and McMaster Universities Arthritis Index (WOMAC) scores, including pain, stiffness, and functional joint motion, in patients with OA compared with the control group (WOMAC-pain, MD = −1.08, CI = 95% [−1.62, −0.53], *I*^2^ = 87%, *P* < 0.05; WOMAC-stiffness, MD = −1.17, CI = 88% [−1.72, −0.63], *I*^2^ = 87%, *P* < 0.05; WOMAC-function, MD = −1.12, CI = 95% [−1.65, −0.58], *I*^2^ = 87%, *P* < 0.05). In addition, subgroup analysis showed that leukocyte-poor (LP) PRP injections were more effective than leukocyte-rich (LR) PRP injections in improving pain symptoms in patients with OA (VAS, LR-PRP, MD = −0.81, CI = 95% [−1.65, −0.03], *I*^2^ = 83%, *P* = 0.06 > 0.05; LP-PRP, MD = −1.62, CI = 95% [−2.36, −0.88], *I*^2^ = 92%, *P* < 0.05). A subgroup analysis based on injection sites showed that no statistical difference in efficacy between intra-articular (IA) combined with intra-osseous (IO) simultaneous PRP injections. IA PRP injections only improved VAS pain scores in patients with OA (IA+IO PRP injections, MD = −0.74, CI =95% [−1.29, −0.18], *I*^2^ = 61%, *P* < 0.05; IA PRP injections, MD = −1.43, CI = 95% [−2.18, −0.68], *I*^2^ = 87%, *P* < 0.05, test for subgroup differences, *P* > 0.05, *I*^2^ = 52.7%).

**Conclusion:**

PRP injection therapy can safely and effectively improve functional activity in patients with OA and produce positive analgesic effects in patients with KOA, TMJOA, and AOA. However, PRP injection therapy did not significantly reduce pain symptoms in patients with HOA. In addition, the analgesic effect of LP-PRP was greater than that of LR-PRP.

**Systematic review registration:**

https://www.crd.york.ac.uk/PROSPERO/, identifier: CRD42022362066.

## 1. Introduction

Osteoarthritis (OA) is a degenerative disease that affects the entire joint and is characterized by progressive cartilage degeneration, synovial inflammation, bone formation, and subchondral sclerosis (1). Clinical features include local inflammation, pain, stiffness, and limited joint movement (2). Currently, OA affects ~3.3–3.6% of the global population and causes moderate-to-severe disability in 43 million people, severely affecting patients' quality of life and increasing the burden on families, making it the 11th most debilitating disease in the world (3). As the population ages and obesity rates rise, the incidence of OA is further increasing (4, 5).

Current OA treatments focus on disease prevention and early treatment (6). Common OA treatments include physical therapy (7), oral medications (8), and intra-articular (IA) injections (9). IA injections, including local anesthetics (10), hyaluronic acid (HA), and corticosteroids (CCS) (11, 12), are often an option for patients with early-to mid-stage OA who fail to achieve satisfactory results after various other non-surgical treatments. However, studies have shown that these pharmacological treatments provide only temporary benefits and are often accompanied by side effects such as stiffness and swelling at the injection site (13). Platelet-rich plasma (PRP) is a more recent treatment that has attracted attention because of its high regenerative capacity, ease of extraction and preparation, low rejection rate, and few adverse effects (14, 15).

PRP is a highly concentrated autologous blood product of platelets and other active substances (16) and may play an important role in autologous cell therapy in various regenerative medicine programs. PRP therapy promotes chondrocyte proliferation and cartilage matrix formation and inhibits the expression of inflammatory factors (17, 18). Inflammation and inflammatory responses are thought to be key factors that cause and accelerate OA development (19). Thus, theoretically, PRP therapy may reduce inflammation during OA treatment.

In recent years, the clinical application of IA PRP injections for OA has been widely promoted; however, the effectiveness of this treatment remains controversial. Many new randomized controlled trials (RCTs) of PRP injection for OA have been published. We conducted a systematic review and meta-analysis of these randomized trials to analyze the efficacy and safety of PRP for OA, with the aim of providing an evidence-based basis for the clinical application of PRP injections for OA treatment.

## 2. Methods

### 2.1. Protocol and registration

Our systematic review was designed and implemented based on the Preferred Reporting Items for Systematic Reviews and Meta-Analyses (PRISMA) guidelines (20). The study was registered in Prospero (CRD42022362066).

### 2.2. Retrieval strategy

In the initial screening, two researchers (XYQ and CG) independently searched Embase, Web of Science, Medline, PubMed, and Cochrane Library databases and collected all studies from the date of database creation to March 2023. The main search terms were “osteoarthritis,” “osteoarthrosis,” “osteoarthritides,” and “platelet-rich plasma.” In addition, we manually searched for other relevant literature, such as studies included in some systematic reviews and meta-analyses, to broaden the search for eligible articles. For instance, the following search strategy was used for PubMed: ((“Osteoarthritis”[Mesh]) OR (((((((Osteoarthritides) OR (Osteoarthrosis)) OR (Osteoarthroses)) OR (Degenerative Arthritides[Title/Abstract])) OR (Degenerative Arthritis[Title/Abstract])) OR (Arthrosis[Title/Abstract])) OR (Arthroses[Title/Abstract]))) AND ((“Platelet-Rich Plasma”[Mesh]) OR ((Plasma, Platelet-Rich) OR (Platelet Rich Plasma))). A similar search strategy was used for the other databases. Full details of the search strategy for all databases can be found in Data Sheet 1.

### 2.3. Inclusion and exclusion criteria

The inclusion and exclusion criteria for literature screening were predetermined to allow for a more rigorous process. Inclusion criteria included (1) patients diagnosed with OA by clinical examination; (2) trial group received PRP injection treatment intervention; (3) control group received HA injection treatment or saline injection treatment; (4) outcome indicators of functional activity and analgesic effects were assessed by relevant scales, such as the Visual Analog Scale (VAS), Western Ontario and McMaster Universities Arthritis Index (WOMAC), Knee Injury and Osteoarthritis Outcome Score (KOOS), and International Knee documentation Committee (IKDC); (5) the experimental design was a RCT; (6) the language was restricted to English.

The exclusion criteria were as follows: (1) animal experiments; (2) IA injection of other drugs, such as Procaine and Lidocaine, over the previous 1 year; (3) full-text content not available; and (4) missing or duplicate experimental data.

### 2.4. Study selection

We imported all retrieved studies into the document management software Endnote 20 and removed duplicate studies. Two researchers (YX and CG) read the title and abstract of each study simultaneously and screened the studies based on previously developed criteria. For further screening, two reviewers downloaded and read the full texts, removed articles that did not meet the inclusion criteria, and discussed them to confirm their eligibility. If there was a disagreement about the screening process for a particular study, a consensus was reached through advice provided by the principal investigator (WL).

### 2.5. Data extraction

Two reviewers (YX and CG) independently extracted the following data from the included literature: first author of the study, year of publication, sample size, patient age, sex, disease type, disease duration, interventions, and outcome indicators of the trial. Specific therapeutic parameters, including the treatment site, dosage, and number of injections, were also recorded in detail for the intervention method. In addition, when the two reviewers (YX and CG) encountered unclear or complicated extraction of complete literature, the original authors were contacted to obtain complete experimental data. After three consecutive emails, the study was considered missing data if no response was received from the original author.

### 2.6. Quality assessment and risk of bias assessment

A quality assessment of the literature was completed independently by two researchers (YX and CG), followed by a discussion to produce consistent results. A risk of bias assessment was conducted using the Cochrane Risk of Bias tool (Review Manager 5.40). Items were assessed in seven areas: blinding of participants and personnel, blinding of outcome assessments, allocation concealment, random sequence generation, incomplete outcome data, selective reporting, and other biases. Risk of bias was graded as high, low, or unclear (21). The risk of bias for each study was documented by mapping the risk of bias assessment.

Heterogeneity between studies was statistically analyzed using RevMan 5.40. The size of heterogeneity was expressed as *I*^2^; high heterogeneity was judged when *I*^2^ ≥ 75%, moderate heterogeneity when 75% > *I*^2^ ≥ 50%, low heterogeneity when 50% > *I*^2^ ≥ 25%, and no heterogeneity when *I*^2^ = 0% (22).

The quality of evidence for the outcome indicators was assessed using the Grading of Recommendations Assessment, Development, and Evaluation (GRADE) system, which examines areas such as study limitations, intermittency, inconsistency, and imprecision of results (23). The results were assessed by grading the evidence for the outcome indicators as “high,” “moderate,” “low,” or “very low,” and the strength of the recommendations was divided into two levels: “strong” and “weak” (24).

### 2.7. Statistical analysis

For statistical and analytical purposes, the extracted study data were entered into RevMan 5.40 software. This meta-analysis was performed using either a fixed-effects or a random-effects model, depending on the size of heterogeneity. When *I*^2^ ≥ 50%, a random effects model was used; when *I*^2^ < 50%, a fixed effects model was used (25). We used 95% confidence intervals (CI) and mean differences (MD) to measure the effect size. The effect sizes of different units of the same outcome index were measured using the standardized mean difference.

## 3. Results

### 3.1. Results of the literature search

**Figure 1:**
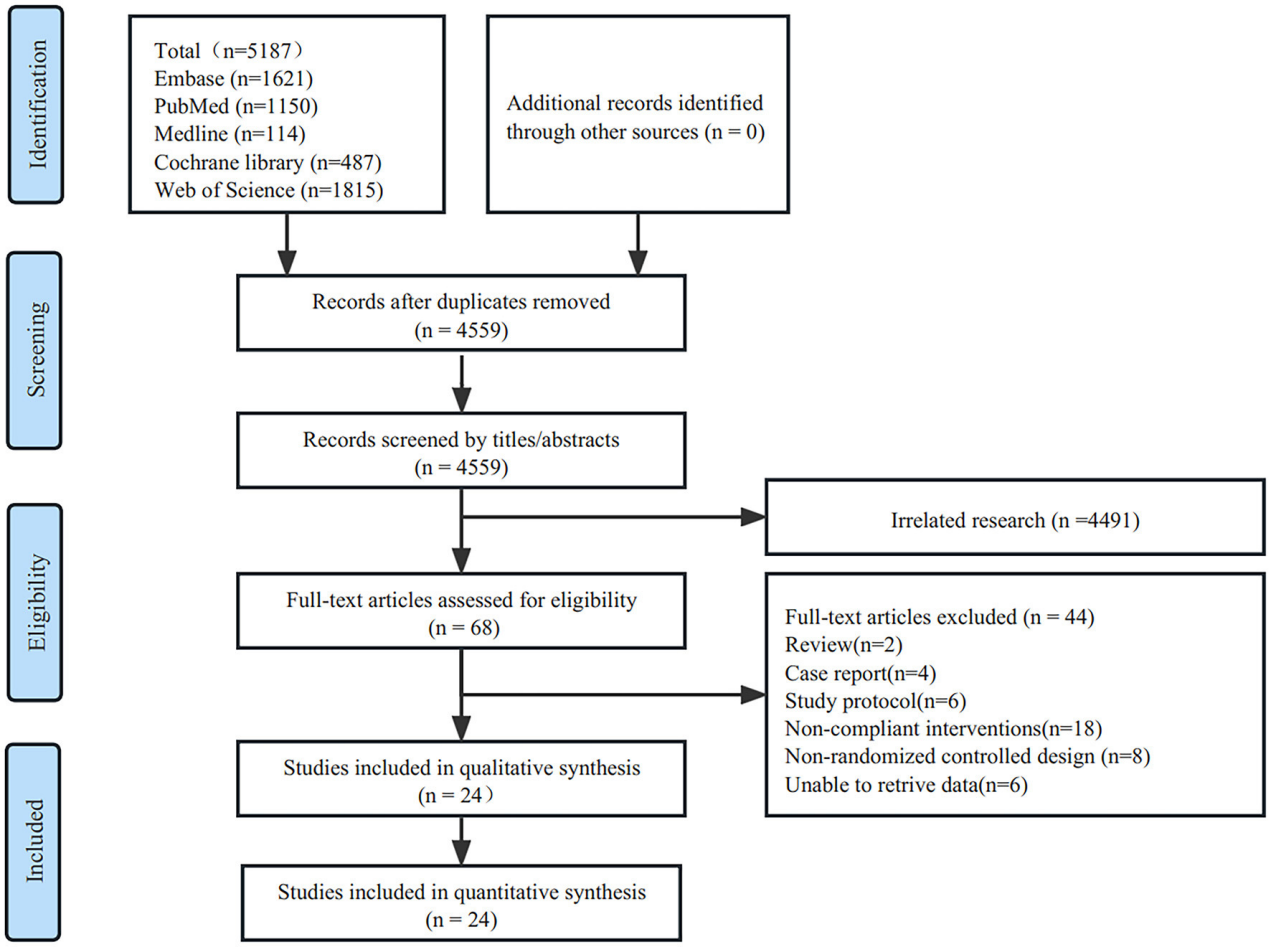
Flow graph of selection and exclusion.

The initial search resulted in 5,187 studies obtained from five databases. After screening for duplicate studies using the software deletion function, 4,559 studies remained. Two reviewers (YQX and CG) read the titles and abstracts of these studies and screened 4,491 studies that were not relevant to the topic. The remaining 68 studies were read, leaving 44 excluded studies, including two reviews, four case reports, six study protocols, 18 studies with non-compliant interventions, 8 non-RCT studies, and 6 studies with no data. Twenty–four eligible studies were included in the final analysis (26–49) (Figure 1).

### 3.2. Characteristics of included studies

Baseline data and experimental intervention parameters were extracted from the 24 included RCTs, summarized in Tables 1, 2, respectively. A total of 1344 patients with OA were eligible across the 24 studies, 712 of whom received PRP injections, and 683 who only received HA or saline as controls (some KOA patients have received treatment in both knees). The intervention characteristics of PRP treatment, such as injection site, injection dose, presence of leukocytes, number of injections, and frequency of injections, are shown in Table 2. In our meta-analysis, 10 studies with a total of 211 patients received one PRP injection, and the remaining 14 studies with a total of 501 patients received two or more PRP injections.

**Table 1:** Study and patient characteristics.

**Study (year)**	**Country**	**Sample Size (n)**	**Sex (M/F)**	**Age (years)**	**Disease type**	**Disease duration**	**Outcomes**	**Adverse events**
Dório et al. (29)	Brazil	G1: 20	1/19	66.4 ± 5.6	KOA	8.4 ± 6.5 years	VAS	Pain
		G2: 21	2/19	62.5 ± 8.1		7.1 ± 6.9 years	WOMAC	
		KOOS				
Bennell et al. (27)	Australia	G1: 144	59/95	62.2 ± 6.3	KOA	5.0 ± 7.1 years	KOOS	pain, swelling and stiffness
		G2: 144	60/84	61.6 ± 6.6		6.0 ± 5.3 years		
Görmeli et al. (45)	Turkey	G1: 39	16/23	53.7 ± 13.1	KOA	>4 months	IKDC	NO
		G2: 40	20/20	52.8 ± 12.8				
Wu et al. (16)	China	G1: 20	5/15	63.3 ± 6.8	KOA	65.4 ± 58.3 months	WOMAC	NO
		G2: 20	60.1 ± 54.6 months					
Lin et al. (31)	China	G1: 31	9/22	61.2 ± 13.1	KOA	> 4 months	WOMAC	NO
		G2: 27	10/17	62.2 ± 11.7			IKDC	
Elik et al. (32)	Turkey	G1: 30	1/29	61.3 ± 7.9	KOA	< 1 year	VAS	NO
		G2: 27	3/24	60.2 ± 6.8		WOMAC		
Barman et al. (33)	India	G1:25	8/17	57.1 ± 4.3	KOA	30.3 ± 12.9 months	VAS	swelling, headache and stiffness
		G2:25	9/16	57.0 ± 5.0		31.5 ± 13.5 months	KOOS	
Nunes et al. (35)	Brazil	G1: 34	4/30	67.6 ± 7.4	KOA	10.3 ± 7.1 years	VAS	NO
		G2: 33	3/30	68.0 ± 6.2		7.8 ± 4.9 years	WOMAC	
Patel et al. (30)	India	G1: 25	5/20	51.6 ± 9.2	KOA	-	VAS	syncope, dizziness and nausea
		G2: 23	6/17	53.7 ± 8.2				
Bastos et al. (37)	Portugal	G1: 9	4/5	60.4 ± 11.3	KOA	-	KOOS	joint effusion, pain
		G2: 9	5/4	54.7 ± 7.2				
Raeissadat et al. (41)	Iran	G1: 21	0/42	57.6 ± 5.9	KOA	< 3 months	VAS	-
		G2: 21					WOMAC	
Ghai et al. (42)	India	G1: 10	5/15	49.8 ± 9.4	KOA	> 4 months	VAS	inflammation, swelling
		G2: 10					WOMAC	
Rayegani et al. (39)	Iran	G1: 31	2/29	58.1 ± 9.0	KOA	> 3 months	WOMAC	pain, swelling and stiffness
		G2: 31		54.7 ± 10.8				
Bastos et al. (37)	Portugal	G1: 16	10/6	60.8 ± 9.9	KOA	-	KOOS	NO
		G2: 14	5/9	55.7 ± 7.8				
Su et al. (34)	China	G1: 27	10/17	50.7 ± 8.7	KOA	> 1 months	VAS	pain, swelling and stiffness
		G2: 25	11/14	54.2 ± 6.6			WOMAC	
Nouri et al. (26)	Iran	G1: 31	8/23	60.3 ± 4.8	HOA	4.3 ± 2.0 months	VAS WOMAC	pain, warmness and heaviness
		G2: 32	10/22		58.2 ± 5.1	4.6 ± 2.5 months		
		G3: 29	7/22		60.9 ± 4.5	3.4 ± 1.5 months		
Cömert Kiliç et al. (28)	Turkey	G1: 18	2/16	32.2 ± 14.3	TMJOA	-	VAS	-
		G2: 12	1/11	35.1 ± 14.8				
Fernández et al. (40)	Spain	G1: 42	2/40	36.7 ± 6.3	TMJOA	> 6 months	VAS	serum extravasation and bleeding
		G2: 50	3/47	34.8 ± 7.9				
Asadpour et al. (44)	Iran	G1: 10	-	29.9 ± 8.5	TMJOA	-	VAS	NO
		G2: 10		29.5 ± 8.9				
		G3: 10		29.5 ± 8.5				
Di Sante et al. (49)	Italy	G1: 21	11/10	71.4 ± 6.0	HOA	-	VAS	-
		G2: 22	9/13	73.6 ± 7.9			WOMAC	
Görmeli et al. (43)	Turkey	G1: 13	5/8	38.6 ± 9.1	AOA	< 24 months	VAS	NO
		G2: 14	8/6	39.7 ± 8.7				
		G3: 13	8/5	40.3 ± 9.4				
Malahias et al. (47)	Greece	G1: 16	3/13	62.8 ± 10.6	TJOA	-	VAS	-
		G2: 16	3/13	63 ± 11.8				
Mei-Dan et al. (48)	Israel	G1: 15	12/3	42.8 ± 18.1	AOA	-	VAS	pain
		G2: 15	11/4	36.5 ± 15.2				
Guney et al. (46)	Turkey	G1:22	11/11	43.9 ± 12.7	AOA	-	VAS	NO
		G2: 19	10/9	37.4 ± 16.0				
		G3: 13	11/2	37.6 ± 15.7				

G1, group 1; G2, group 2; G3, group 3; n, number; M, male; F, female; KOA, knee osteoarthritis; HA, hip osteoarthritis; AOA, ankle osteoarthritis; TMJOA, temporomandibular joint osteoarthritis; TJOA, the thumb or trapeziometacarpal joint osteoarthritis; VAS, Visual Analog Scale; WOMAC, Western Ontario McMaster Universities Osteoarthritis Index; KOOS, Knee Injury and Osteoarthritis Outcome Score; IKDC, International Knee Documentation Committee; NO, no adverse events; “-”, not report.

**Table 2:** Therapeutic parameters of included RCTs.

**Study(year)**	**Injection site**	**Injection volume (ml)**	**LP-PRP or LR-PRP**	**Number of injections**	**Injection time**
Dório et al. (29)	IA	1.4 ~ 5 ml	LP	2	Once every 2 weeks, 4 weeks
Bennell et al. (27)	IA	5 ml	LP	3	Once a week, 3 weeks
Görmeli et al. (45)	IA	5 ml	-	3	Once a week, 3 weeks
Wu et al. (16)	IA	4 ml	LR	1	-
Lin et al. (31)	IA	2 ml	LP	3	Three times a week, 1 week
Elik et al. (32)	IA	4 ml	-	3	Once a week, 3 weeks
Barman et al. (33)	IO + IA	10 ml + 8 ml	-	1	-
	IA	8 ml	-	-	
Nunes et al. (35)	IA	-	-	1	-
Patel et al. (30)	IA	8 ml	LP	2	Once every three weeks, 6 weeks
Bastos et al. (37)	IA	-	-	1	-
Raeissadat et al. (41)	IA	-	LR	2	Once a month, 2 months
Ghai et al. (42)	IA	8 ml	-	1	-
Rayegani et al. (39)	IA	4 ~ 6 ml	LR	2	Once a month, 2 months
Bastos et al. (37)	IA	-	LP	1	-
Su et al. (34)	IO + IA	2 ml + 2 ml	LR	2	Once every 2 weeks, 4 weeks
	IA	6 ml	-	-	
Nouri et al. (26)	IA	5 ml	-	2	Once every 2 weeks, 4 weeks
Cömert Kiliç et al. (28)	IA	-	-	5	Five times a month, 1 month
Fernández et al. (40)	IA	5 ml	LP	1	-
Asadpour et al. (44)	IA	1 ml	-	1	-
Di Sante et al. (49)	IA	3 ml	-	3	Once a week, 3 weeks
Görmeli et al. (43)	IA	-	-	1	-
Guney et al. (46)	IA	4 ml	-	1	-
Malahias et al. (47)	IA	2 ml	-	2	Once every 2 weeks, 4 weeks
Mei-Dan et al. (48)	IA	2 ml	-	3	Once every 2 weeks, 4 weeks

IA, intra-articular; IO, intra-osseous; LR-PRP, Leukocyte-Rich Platelet-Rich Plasma; LP-PRP, Leukocyte-Poor Platelet-Rich Plasma; “-”, not report.

### 3.3. Results of the quality assessment

**Figure 2:**
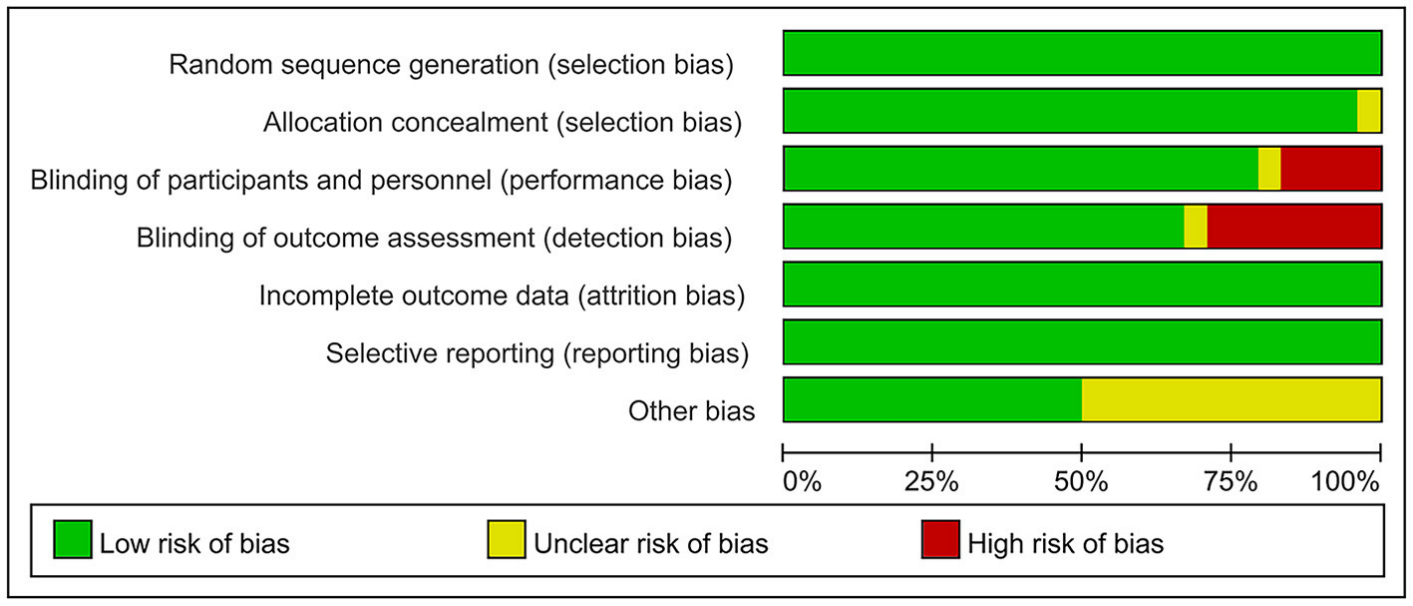
Risk of bias graph: judgements about each risk of bias item presented as percentages.

**Figure 3:**
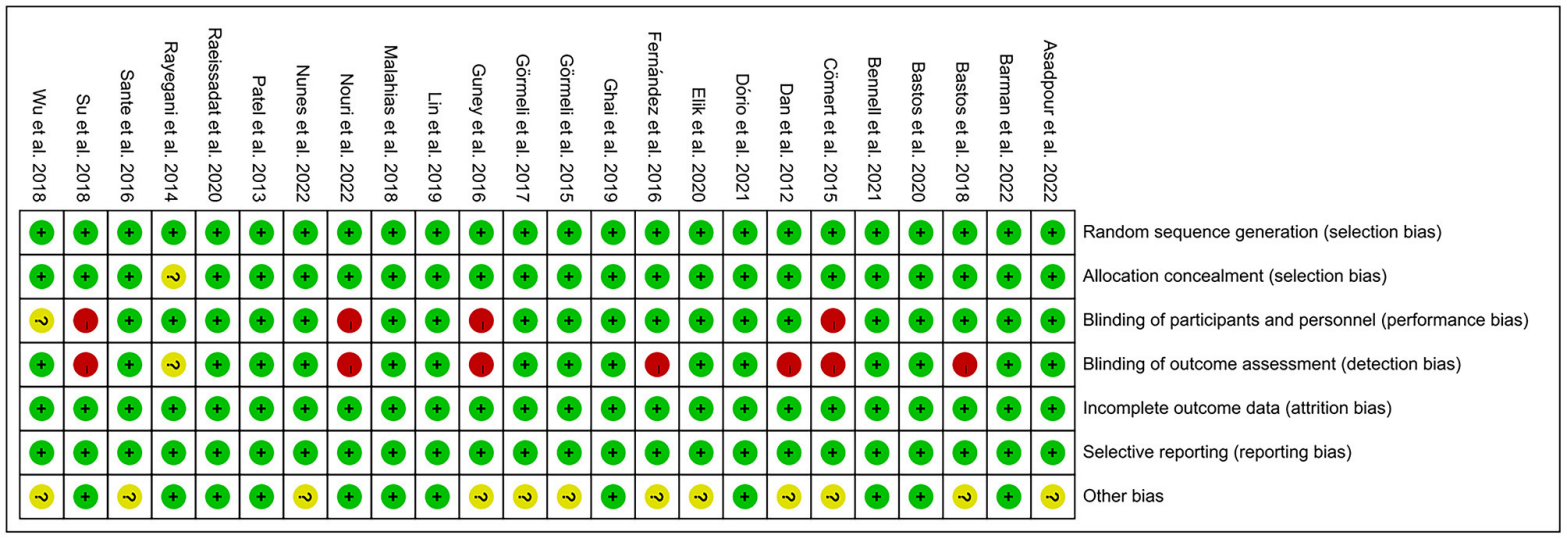
Risk of bias summary: judgements about each risk of bias item for each included study.

The risk of bias was assessed using the Cochrane Risk of Bias Assessment Tool (RevMan 5.40) for the 24 included RCTs. In 4 of the 24 studies, investigators did not use reasonable blinding of participants and staff, resulting in a high risk of performance bias. In another 7 of the 24 studies, investigators did not use reasonable blinding when assessing outcome indicators, resulting in a high risk of detection bias. Other studies have shown a low or unclear risk in all risk–bias assessments. Overall, the included RCTs had a low risk of bias (Figures 2, 3).

We assessed the level of evidence for the main outcome indicators (VAS, IKDC, KOOS, and WOMAC) in the included studies by using GRADE. The quality of evidence for IKDC and WOMAC was assessed as moderate owing to high heterogeneity (*I*^2^ > 80%), with serious inconsistencies between studies. The quality of evidence for the VAS and KOOS was rated as high because no serious risk or downgrading factors were identified in any of the projects. Overall, the GRADE recommendation rating was “strong” for the four primary outcome indicators (Table 3).

**Table 3:** Grading of recommendations assessment, development, and evaluation (GRADE) quality of evidence.

**Outcome**	**Number of studies**	**Design**	**Study limitations**	**Inconsistency**	**Indirectness**	**Imprecision**	**Publication bias**	**Effect size**	**GRADE quality**	**Symbolic expression**
VAS	17	RCT	0	−1^*^	0	0	0	0	Moderate	⊕⊕⊕⊖
IKDC	2	RCT	0	0	0	0	0	0	High	⊕⊕⊕⊕
KOOS	5	RCT	0	0	0	0	0	0	High	⊕⊕⊕⊕
WOMAC	11	RCT	0	−^*^	0	0	0	0	Moderate	⊕⊕⊕⊖

VAS, Visual Analog Score; IKDC, International Knee Documentation Committee Subjective Knee Form; KOOS, Knee Injury and Osteoarthritis Outcome Score; WOMAC, Western Ontario and McMaster University Osteoarthritis Index.

* Heterogeneity is too high (I^2^ > 80%).

### 3.4. Results of statistical analysis

**Figure 4:**
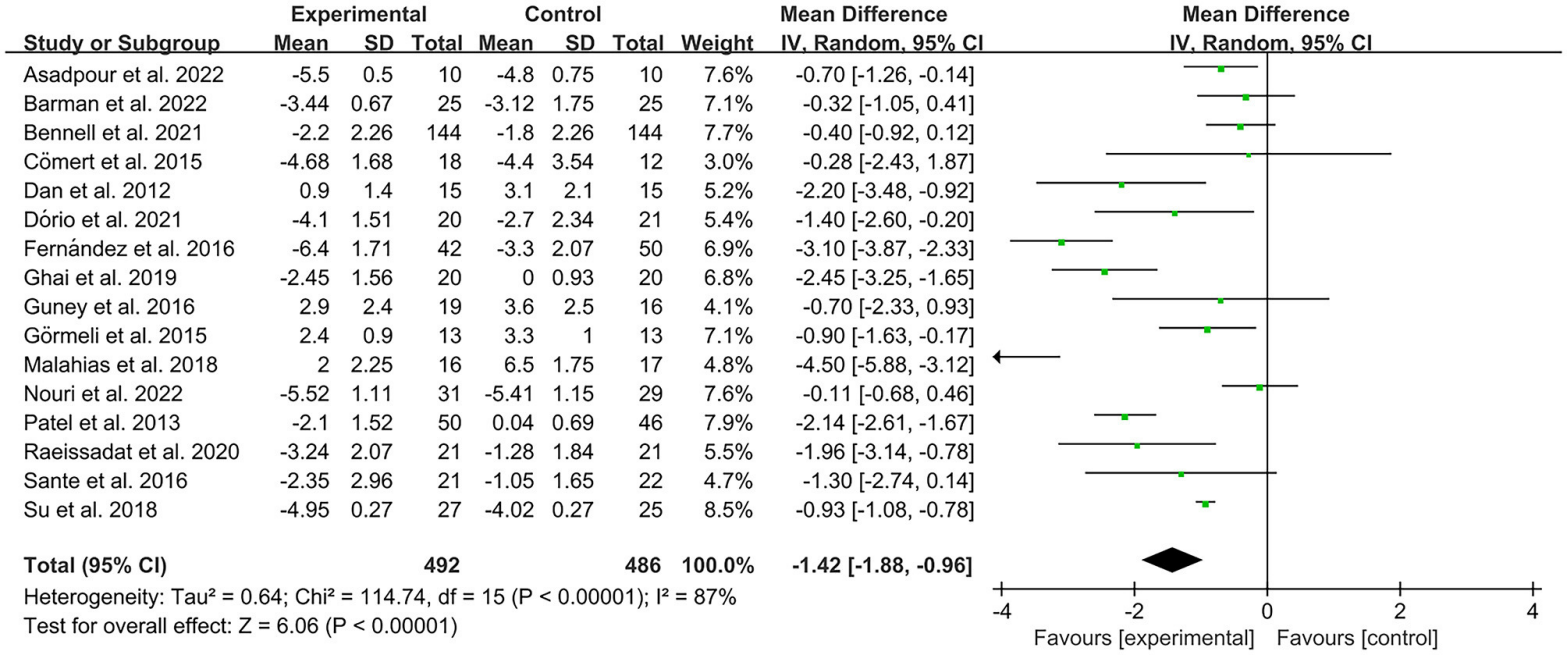
Forest plot for the VAS score.

**Figure 5:**
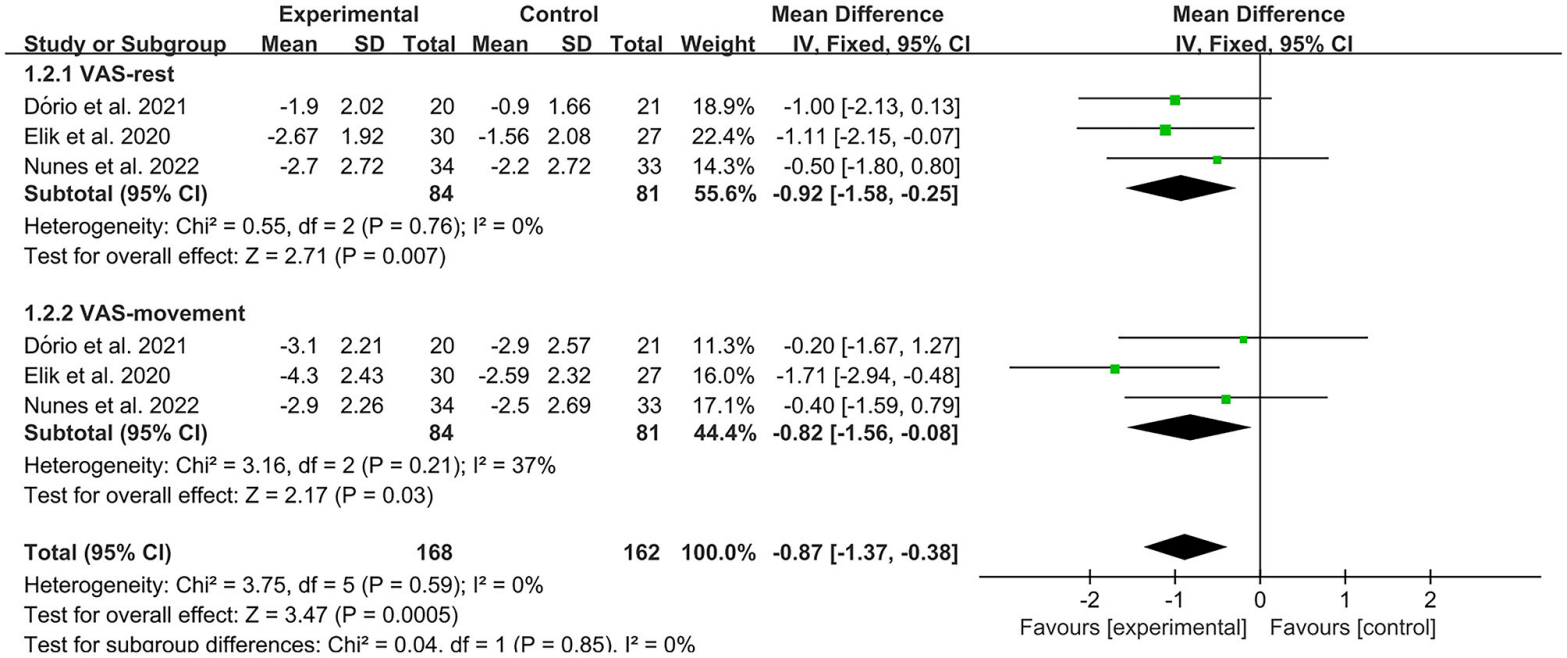
Subgroup analysis for the VAS scores at different types.

**Figure 6:**
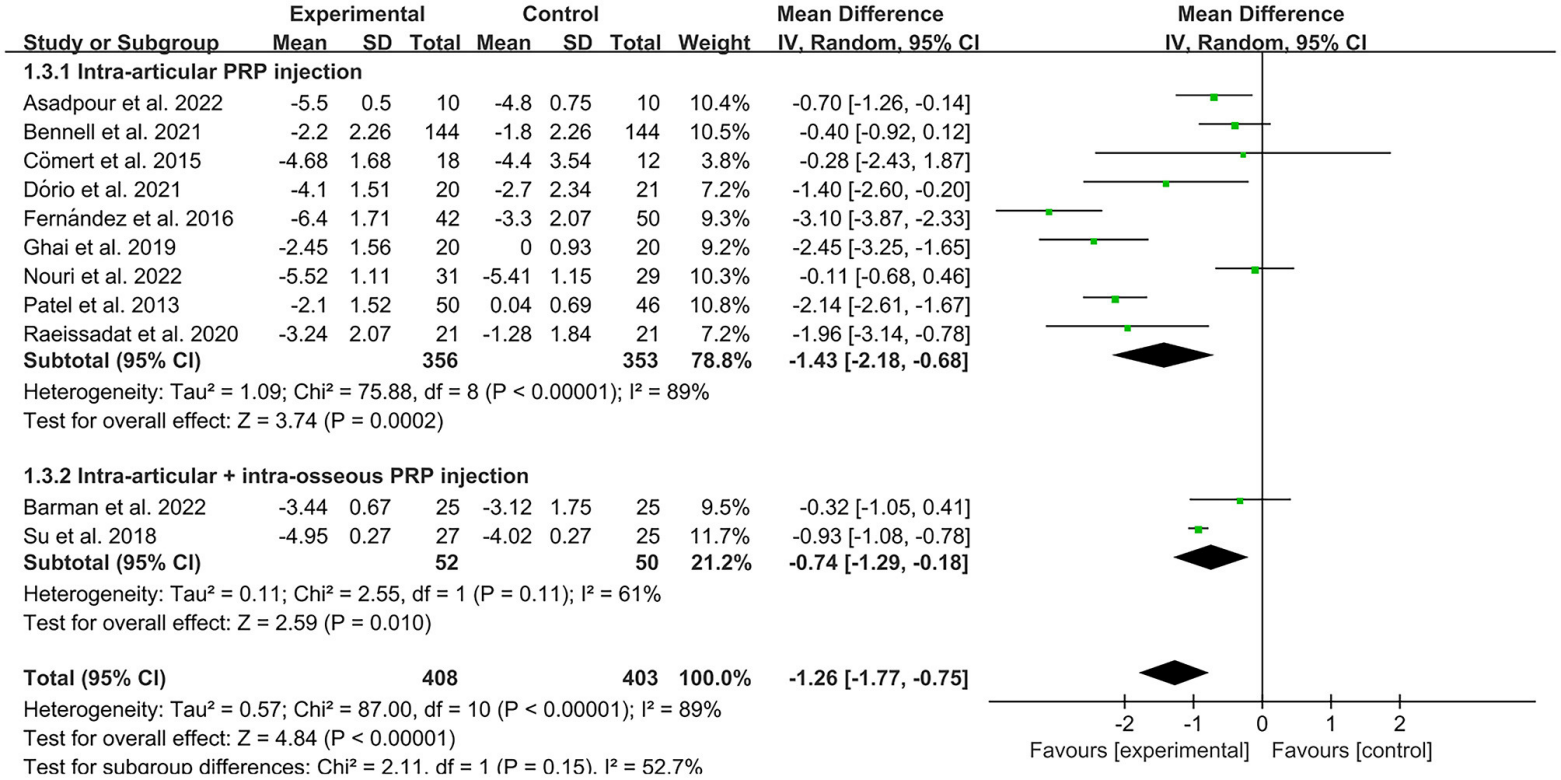
Subgroup analysis for the VAS scores at different injection sites.

**Figure 7:**
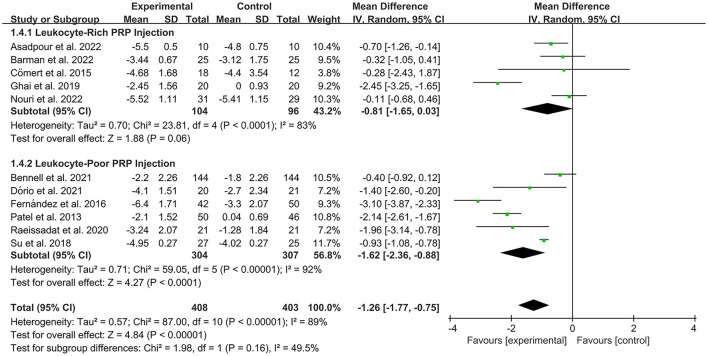
Subgroup analysis for the VAS scores at different leukocyte concentrations.

The VAS was used by investigators in 17 studies (26, 28–30, 32–35, 40–42, 44–49) to assess pain intensity. In these studies, 966 subjects participated in PRP injection therapy trials, divided into experimental and control groups. The *I*^2^ value was 87%. Therefore, a random-effects model was used. Data analysis of the forest plot showed that the experimental group with PRP injection as an intervention had a significant improvement in VAS OA patients' pain scores compared to the control group (MD = −1.42, CI = 95% [−1.88, −0.96], *I*^2^ = 87%, *P* < 0.05; Figure 4). A subgroup analysis showed that the pain efficacy of PRP in patients with OA was synchronized in both the rest and movement states, and pain relief was consistent in both states (VAS-rest, MD = −0.92, CI = 95% [−1.58, −0.25], *I*^2^ = 89%, *P* < 0.05; VAS-movement, MD = −0.82, CI = 95% [−1.56, −0.08], test for subgroup differences, *P*>0.05, *I*^2^= 0%; Figure 5). Additional subgroup analyses based on injection sites showed no statistical difference in efficacy between IA combined with intra-osseous (IO) simultaneous PRP injections and IA PRP injections in improving VAS scores in patients with OA (IA+IO PRP injections, MD= −0.74, CI = 95% [−1.29, −0.18], *I*^2^ = 61%, *P* < 0.05; IA PRP injections, MD= −1.43, CI = 95% [−2.18, −0.68], *I*^2^ = 87%, *P* < 0.05, test for subgroup differences, *P* > 0.05, *I*^2^ = 52.7%; Figure 6). A subgroup analysis based on leukocyte concentration showed that leukocyte-rich (LR) PRP injections were not considered effective for patients' pain symptoms. In contrast, leukocyte-poor (LP) PRP injections were effective in relieving pain (VAS, LR-PRP, MD = −0.81, CI = 95% [−1.65, −0.03], *I*^2^ = 83%, *P* = 0.06 > 0.05; LP-PRP, MD = −1.62, CI = 95% [−2.36, −0.88], *I*^2^ = 92%, *P* < 0.05; test for subgroup differences, *P*>0.05, *I*^2^ = 11.6%; Figure 7).

**Figure 8:**
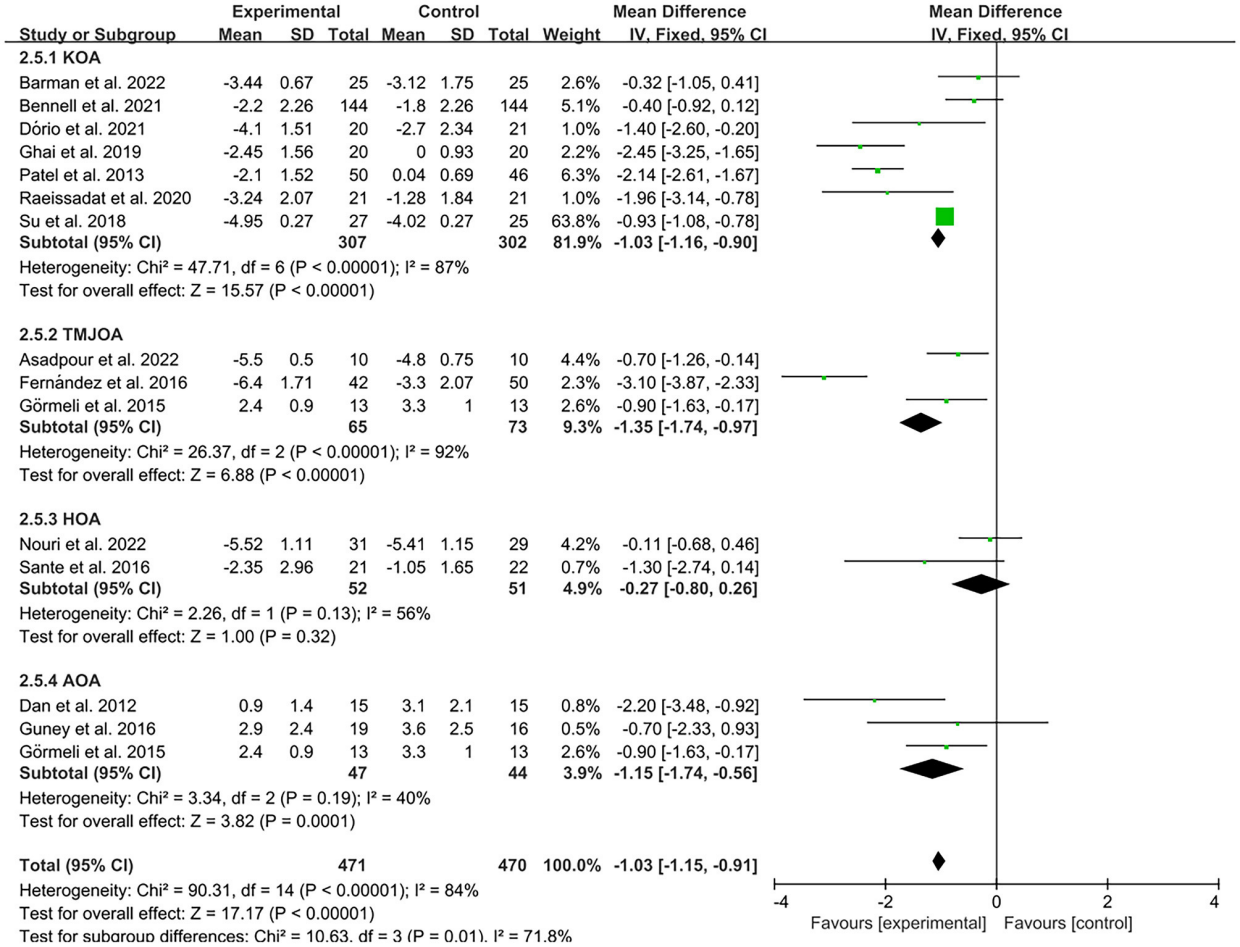
Subgroup analysis for the VAS scores at different types of osteoarthritis.

The results of the forest plot data analysis based on PRP treatments of different joint sites indicate that PRP injection therapy was effective in reducing VAS pain scores in patients with ankle osteoarthritis (AOA), knee osteoarthritis (KOA) and temporomandibular joint osteoarthritis (TMJOA) compared to controls (AOA, MD = −1.15, CI = 95% [−1.74, −0.56], *I*^2^ = 40%, *P* < 0.05; KOA, MD = −1.03, CI = 95% [−1.16, −0.9], *I*^2^ = 87%, *P* < 0.05; TMJOA, MD = −1.35, CI = 95% [−1.74, −0.97], *I*^2^ = 92%, *P* < 0.05; Figure 8). However, there was no statistically significant difference between the experimental and control groups in VAS pain scores of hip osteoarthritis (HOA, MD = −0.27, CI = 95% [−0.8, 0.26], *I*^2^ = 56%, *P* > 0.05; Figure 8).

**Figure 9:**
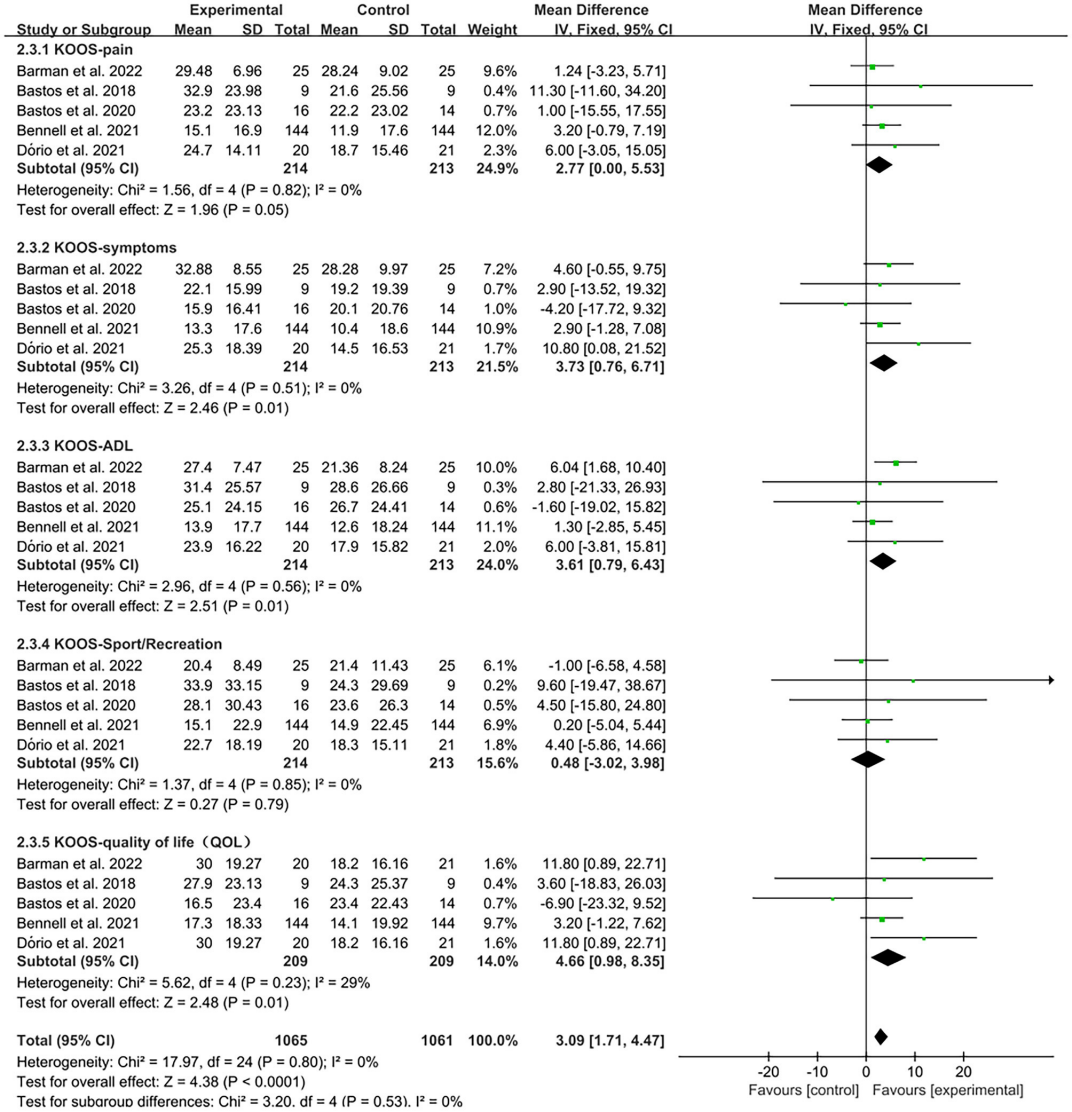
Subgroup analysis for the KOOS scores at different types.

The KOOS was used by investigators in five studies (27, 29, 33, 37, 38) to assess treatment effects. In these studies, 427 subjects participated in PRP injection treatment trials, divided into experimental and control groups. The *I*^2^ value was calculated to be 0%. Therefore, we used a fixed effects model in this study. Forest plots with KOOS as an outcome indicator showed that the experimental group had significantly improved pain scores, symptom scores, activities of daily living scale (ADL) scores, and Quality of Life scores (QOL) in patients with OA compared with the control group, but no significant improvement was observed in the sport scores (KOOS-pain, MD = 2.77, CI = 95% [0, 5.53], *I*^2^ = 0%, *P* < 0.05; KOOS-symptoms, MD = 3.73, CI = 95% [0.76, 6.71], *I*^2^ = 0%, *P* < 0.05; KOOS-ADL, MD = 3.61, CI = 95% [0.79, 6.43], *I*^2^ = 0%, *P* < 0.05; KOOS-QOL, MD = 4.66, CI = 95% [0.98, 8.35], *I*^2^ = 29%, *P* < 0.05; KOOS-sport, MD = 0.48, CI = 95% [−3.02, 3.98], *I*^2^ = 0%, *P* > 0.05; Figure 9).

**Figure 10:**
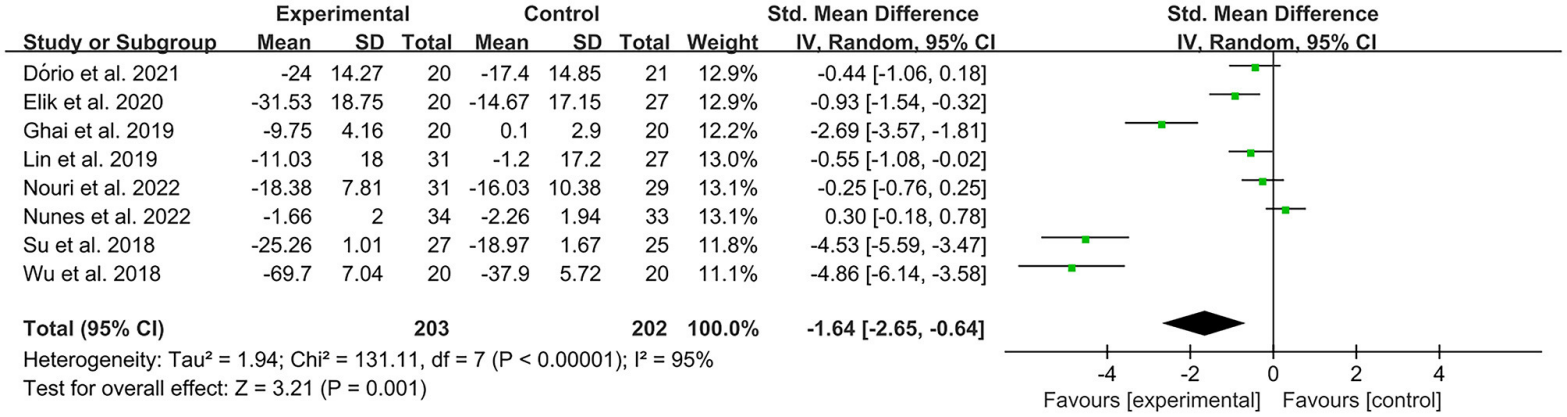
Forest plot for the WOMAC score.

**Figure 11:**
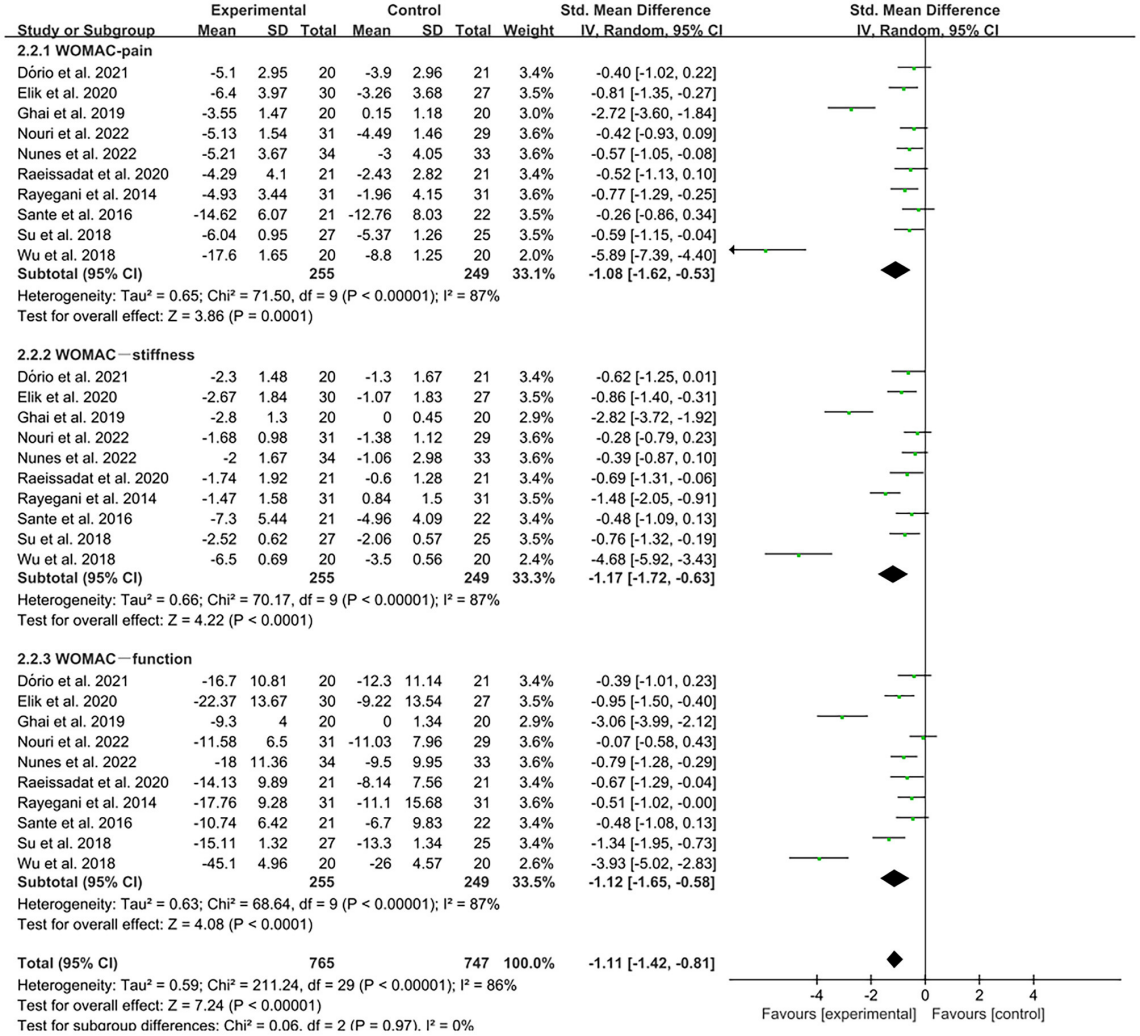
Subgroup analysis for the WOMAC scores at different types.

The WOMAC was used by investigators in 11 studies (26, 29, 31, 32, 34–36, 39, 41, 42, 49) to assess joint functional activity. In these studies, 574 subjects participated in PRP injection treatment trials, divided into experimental and control groups. The *I*^2^ value was 95%. Therefore, a random-effects model was used. The forest plots with WOMAC as an outcome indicator showed that WOMAC scores of patients with OA improved in the experimental group but not the control group (MD = −1.64, CI = 95% [−2.65, −0.64], *I*^2^ = 95%, *P* < 0.05; Figure 10). In the experimental group, patients showed significant improvements in joint pain, joint stiffness, and functional activity (WOMAC-pain, MD = −1.08, CI = 95% [−1.62, −0.53], *I*^2^ = 87%, *P* < 0.05; WOMAC-stiffness, MD = −1.17, CI = 88% [−1.72, −0.63], *I*^2^ = 87%, *P* < 0.05; WOMAC- function, MD = −1.12, CI = 95% [−1.65, −0.58], *I*^2^ = 87%, *P* < 0.05; Figure 11).

**Figure 12:**
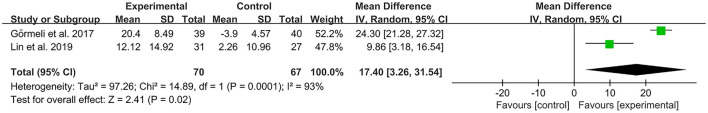
Forest plot for the IKDC score.

The IKDC was used by investigators in two studies (31, 43) to assess the functional activity of the joints. In these studies, 137 subjects participated in PRP injection treatment trials and were divided into experimental and control groups. The *I*^2^ value was 93%. Therefore, a random-effects model was used. The results of the forest plot data analysis with IKDC as an outcome indicator showed that the experimental group receiving PRP demonstrated improved OA patient IKDC scores when compared to the control group (MD = 17.4, CI = 95% [3.26, 31.54], *I*^2^ = 93%, *P* < 0.05; Figure 12).

### 3.5. Adverse event reporting results

Of the 24 studies included, 11 reported adverse reactions after PRP injections in patients with OA; however, most symptoms were mild and transient, such as pain and swelling at the injection site. Of these 11 studies, 2 showed more adverse reactions in the PRP injection group, with patients presenting with syncope, dizziness, headache, nausea, gastritis, sweating, and tachycardia, in addition to pain and stiffness (26, 30). Only one study reported intense knee pain in two patients, and all adverse events reported were resolved without any sequelae (38). Based on the results reported in all studies, a few patients withdrew from the experimental studies because they experienced excessive adverse effects (34).

## 4. Discussion

We analyzed PRP treatment of OA and also discussed the intervention parameters, such as PRP injection sites and leukocyte concentration contained in the injection solution, hoping to provide reference suggestions for the optimal treatment protocol of clinical PRP.

Currently, PRP is widely used for wound healing and tissue regeneration in orthopedics, dentistry, and plastic surgery. It has been shown to promote angiogenesis, cell proliferation, and collagen synthesis to repair tendons, ligaments, cartilage, and other avascular damaged tissues with low self-healing capacity; to act as an anti-inflammatory and analgesic; and to improve motor function (50–52). How PRP exerts its efficacy in the treatment of OA is not fully understood, but numerous studies have shown that it may be related to the presence of various growth factors and inflammatory cells. Platelets contain a large number of α particles, which include a variety of bioactive factors: transforming growth factor (TGF)-β, platelet-let-derived growth factor (PDGF), vascular endothelial growth factor (EGF), hepatocyte growth factor, epidermal growth factor, fibroblast growth factor, and insulin-like growth factor (53, 54). In OA treatment, within 10 min after the injection of PRP activated by exogenous activators, platelets aggregate and coagulate in the joint cavity, and within 1 h nearly 95% of α particles secrete large amounts of cytokines and growth factors (55). After the platelets are activated, these bioactive factors are released into the bloodstream and play an important role in tissue repair and regeneration. TGF-β and PDGF are important bioactive factors that promote tissue repair (56). Interleukin (IL)-1β interferes with the normal metabolic activity of chondrocytes, inhibits normal chondrocyte differentiation and induces chondrocyte apoptosis, while TGF-β inhibits the interference of IL-1β with chondrocytes and prevents chondrocyte apoptosis (57). TGF-β further activates activinreceptor-like kinases-5 (ALK-5), which regulates cartilage terminal differentiation through the Smad signaling pathway, has a role in promoting chondrocyte proliferation and differentiation, and induces bone marrow mesenchymal stem cells (BMSCs) to differentiate into chondrocytes, regulate the proliferation of other growth factors, and inhibit the expression of some inflammatory factors. PDGF promotes osteoblast proliferation and chemotaxis, enhances collagen synthesis, and stimulates fibroblast proliferation and chemotaxis (58). PRP contains various plasma proteins that activate fibrinogen to form fibrin scaffolds, induce chondrocyte proliferation and differentiation, and promote cartilage damage repair (59).

Most current studies on PRP have used IA injections, with some investigators studying injection sites to determine optimal injection locations. For example, subchondral IO PRP injections have become a popular research topic. The subchondral bone, the bony component of the distal end of the calcified cartilage, is located below the mineralized zone of the articular cartilage and forms the articular cartilage-subchondral bone complex unit with the cartilage. IO PRP injections, which target the subchondral bone to promote recovery of the subchondral bone, articular cartilage, and synovium, are effective in treating severe degenerative lesions (60). In the treatment of KOA, IO injection of PRP directly reaches the subchondral bone, maintains the tissue and BMSCs in the PRP matrix, promotes subchondral bone repair, and modulates the inflammatory environment, thus slowing the progression of KOA, and possibly even having a direct impact on stopping the progression of KOA (61). Several studies have shown that IO PRP injections are effective in improving the symptoms of OA (60, 62), and two of the included studies used “IA+IO” (intra-articular combined with intra-osseous) injections. Interestingly, according to our subgroup analysis, we found no statistical difference between the “IA+IO” group and the IA group improving VAS pain scores in patients with OA, probably due to the small sample size (*n* < 100) and the fact that we only analyzed differences in pain. Further comparative studies are needed to collect data from more studies for functional improvement as well as disease progression.

Further, many types of PRP injections are currently available. Dohan Ehrenfest et al. (63) first proposed to classify PRP products into the following four categories based on their leukocyte and fibrin content: (1) leukocyte-poor or pure platelet-rich plasma (LP-PRP) containing high concentrations of platelets with little or no leukocytes; (2) leucocyte- and platelet-rich plasma (LR-PRP) with a high concentration of platelets and a large number of leukocytes; (3) leucocyte-poor or pure platelet-rich fibrin (PPRF) with rich circulating fibrin with little or no leukocytes; and (4) leucocyte- and platelet-rich fibrin (L-PRF), rich in circulating fibrin and a large number of leukocytes. Two low-density fibrin formulations, LP-PRP and LR-PRP, are often used in injectable therapy for osteoarthritic disease. P-PRF and L-PRF tend to be gel-like because of their high fibrin content. The role of leukocytes in PRP is currently controversial, and some studies have suggested that LR-PRP and L-PRF exert anti-inflammatory and antioxidant effects, promote chondrocyte proliferation, inhibit matrix calcification, and have good prospects for the treatment of OA (18). However, a large body of high-quality evidence has recently emerged to support the use of LP-PRP in OA treatment (54, 64). Based on our subgroup analysis, it cannot yet be assumed that LR-PRP injections are effective in treating pain; therefore, we analyzed the characteristics of the leukocytes themselves. Because leukocytes may produce matrix metalloproteinase-1 (MMP-1) and inflammatory cytokines that are detrimental to joint inflammation and pain (65), injections of LR-PRP cause a more severe acute inflammatory response and increased synovial cell death (66–68), in addition to an increased risk of local adverse effects, pain, and swelling (69). Therefore, in clinical applications, LP-PRP treatment is more effective in improving pain symptoms in OA patients.

In addition, among the subjects included in the current study, only patients with HOA did not show significant efficacy after PRP injection compared to the control group. This may be due to a number of reasons. First, the hip joint itself differs from the other three joints in that it has a deeper joint cavity, fewer blood vessels in the joint cavity, and is prone to femoral head necrosis, thus making it more difficult to perform PRP injection therapy, resulting in the inability of PRP to fully function in the hip cavity. However, it has been argued that the current inclusion of insufficient RCTs with small sample sizes (*n* < 100) may result in statistical errors. To justify this result, multicenter RCTs of PRP injections for HOA with a larger sample size are required.

## 5. Limitations

This meta-analysis has several limitations. First, we included only two RCTs in which the subjects were patients with HOA, and the small sample size (*n* < 100) tended to bias the assessment of treatment effects, thus affecting the results of the analysis. Second, there were too few functional activity assessment indicators for patients with OA; the main outcome indicators were focused on patients with KOA, and the assessment results were not yet fully representative of the degree of functional activity improvement in OA patients. Third, although our study showed a positive therapeutic effect of LP-PRP injection therapy in OA patients, no follow-up records were reported to further confirm the long-term effect of PRP injection. Finally, there were differences in PRP injection concentrations and injection doses between RCTs; however, the current data do not allow for a subgroup analysis of these intervention parameters. Therefore, more multicenter, follow-up, double-blind, RCTs should be conducted in the future to allow for longitudinal and cross-sectional comparisons under different PRP intervention parameters to explore the optimal treatment protocol for PRP injection and improve the clinical efficacy of PRP injections in patients with OA.

## 6. Conclusion

This meta-analysis indicated that PRP injections were effective in reducing pain symptoms in patients with KOA, TMJ, and AOA but did not show significant efficacy in patients with HOA. Compared to LR-PRP, LP-PRP injection therapy was more effective in improving pain symptoms in OA patients. In addition, PRP injection therapy can effectively improve the functional activity of OA patients and has a high level of safety for clinical applications.

## Data availability statement

The original contributions presented in the study are included in the article/Supplementary material, further inquiries can be directed to the corresponding authors.

## Author contributions

Conceived, designed, and drafted the manuscript: YX, CG, and XP. Critical revision: XP, XL, XS, and XT. Data collection and analysis: YX and CG. Supervised the work: WL and YW. Final approval of the article: WL, YW, and YL. All authors contributed to the article and approved the submitted version.
