# Supplementary material for: Efficacy and safety of platelet-rich plasma injections for the treatment of osteoarthritis: a systematic review and meta-analysis of randomized controlled trials
Source: Front Med (Lausanne). 2023 Jun 27;10:1204144. doi: 10.3389/fmed.2023.1204144 (PMC10333515; doi:10.3389/fmed.2023.1204144)
Supplement: Supplementary file 1 [file Data_Sheet_1.DOCX]

**Search Strategies for All Databases**

**PubMed:**

#1：**"Osteoarthritis"[Mesh]**

#2：**((((((Osteoarthritides) OR (Osteoarthrosis)) OR (Osteoarthroses)) OR (Degenerative Arthritides[Title/Abstract])) OR (Degenerative Arthritis[Title/Abstract])) OR (Arthrosis[Title/Abstract])) OR (Arthroses[Title/Abstract])**

#3：#1 **OR** #2

**("Osteoarthritis"[Mesh]) OR (((((((Osteoarthritides) OR (Osteoarthrosis)) OR (Osteoarthroses)) OR (Degenerative Arthritides[Title/Abstract])) OR (Degenerative Arthritis[Title/Abstract])) OR (Arthrosis[Title/Abstract])) OR (Arthroses[Title/Abstract]))**

#4：**"Platelet-Rich Plasma"[Mesh]**

#5：**(Plasma, Platelet-Rich) OR (Platelet Rich Plasma)**

#6：#4 **OR** #5

**("Platelet-Rich Plasma"[Mesh]) OR ((Plasma, Platelet-Rich) OR (Platelet Rich Plasma))**

#7：#3 **AND** #6

**(("Osteoarthritis"[Mesh]) OR (((((((Osteoarthritides) OR (Osteoarthrosis)) OR (Osteoarthroses)) OR (Degenerative Arthritides[Title/Abstract])) OR (Degenerative Arthritis[Title/Abstract])) OR (Arthrosis[Title/Abstract])) OR (Arthroses[Title/Abstract]))) AND (("Platelet-Rich Plasma"[Mesh]) OR ((Plasma, Platelet-Rich) OR (Platelet Rich Plasma)))**

("Osteoarthritis"[MeSH Terms] OR ("Osteoarthritis"[MeSH Terms] OR "Osteoarthritis"[All Fields] OR "osteoarthritides"[All Fields] OR ("Osteoarthritis"[MeSH Terms] OR "Osteoarthritis"[All Fields] OR "osteoarthrosis"[All Fields]) OR ("Osteoarthritis"[MeSH Terms] OR "Osteoarthritis"[All Fields] OR "osteoarthroses"[All Fields]) OR "degenerative arthritides"[Title/Abstract] OR "degenerative arthritis"[Title/Abstract] OR "Arthrosis"[Title/Abstract] OR "Arthroses"[Title/Abstract])) AND ("Platelet-Rich Plasma"[MeSH Terms] OR ("Platelet-Rich Plasma"[MeSH Terms] OR ("platelet rich"[All Fields] AND "plasma"[All Fields]) OR "Platelet-Rich Plasma"[All Fields] OR ("plasma"[All Fields] AND "platelet"[All Fields] AND "rich"[All Fields]) OR "plasma platelet rich"[All Fields] OR ("Platelet-Rich Plasma"[MeSH Terms] OR ("platelet rich"[All Fields] AND "plasma"[All Fields]) OR "Platelet-Rich Plasma"[All Fields] OR ("platelet"[All Fields] AND "rich"[All Fields] AND "plasma"[All Fields]) OR "Platelet-Rich Plasma"[All Fields])))

**Embase:**

#1：**'osteoarthritis'/exp OR 'osteoarthritis' OR 'osteoarthritides'**

#2：**'osteoarthrosis'/exp OR 'osteoarthrosis' OR 'osteoarthroses'**

#3：#1 **OR** #2

#4：**'platelet-rich plasma' OR 'plasma, platelet-rich' OR 'platelet rich plasma'**

#5：#3 **AND** #4

**Cochrane Library:**

#1：**MeSH descriptor: [Osteoarthritis] explode all trees**

#2：**(Osteoarthritis):ti,ab,kw OR (Osteoarthritides):ti,ab,kw OR (Osteoarthrosis):ti,ab,kw OR (Osteoarthroses):ti,ab,kw**

#3：**(Arthrosis):ti,ab,kw OR (Arthroses):ti,ab,kw OR (Osteoarthrosis Deformans):ti,ab,kw**

#4：**(Degenerative Arthritides):ti,ab,kw OR (Degenerative Arthritis):ti,ab,kw**

#5：#1 **OR** #2 **OR** #3 **OR** #4

#6：**MeSH descriptor: [Platelet-Rich Plasma] explode all trees**

#7：**(Plasma, Platelet-Rich):ti,ab,kw OR (Platelet Rich Plasma):ti,ab,kw OR (platelet-rich plasma):ti,ab,kw**

#8：#6 **OR** #7

#9：#5 **AND** #8

**Ovid MEDLINE**

#1：***Osteoarthritis/**

#2：**Osteoarthritides.mp.**

#3：**Osteoarthroses.mp.**

#4：**Osteoarthrosis.mp.**

#5：#1 **OR** #2 **OR** #3 **OR** #4

#6：***Platelet-Rich Plasma/**

#7：**Plasma, Platelet-Rich.mp.**

#8：**Platelet Rich Plasma.mp.**

#9：#6 **OR** #7 **OR** #8

#10：#5 **AND** #9

**Web of Science:**

#1：**((TS=(Osteoarthritis)) OR TS=(Osteoarthritides)) OR TS=(Osteoarthroses)**

#2：**((((TS=(Degenerative Arthritides)) OR TS=(Degenerative Arthritis)) OR TS=(Arthrosis)) OR TS=(Arthroses)) OR TS=(Osteoarthrosis Deformans)**

#3：#1 **OR** #2

#4：**((TS=(platelet-rich plasma)) OR TS=(PRP)**

#5：**#3 AND #4**
